# Echocardiographic Markers of Cardiac Response to Therapy in Patients with Light Chain Amyloidosis

**DOI:** 10.1111/echo.70428

**Published:** 2026-03-25

**Authors:** Daniel Ng, Stephanie Wu, Huiyan Ma, Sarah Lee, Carlos Gomez Luna, James Sanchez, Michael Rosenzweig, Faizi Jamal

**Affiliations:** ^1^ City of Hope Lennar Foundation Cancer Center Irvine California USA; ^2^ City of Hope Duarte Cancer Center Duarte California USA

**Keywords:** cardiac response, global longitudinal strain, left atrial strain, light chain cardiac amyloidosis, right ventricular strain

## Abstract

**Purpose:**

The aim of this study was to identify echocardiographic parameters which correlate with cardiac response to treatment in patients with light chain cardiac amyloidosis (AL‐CA).

**Methods:**

We identified 39 patients with AL‐CA treated at City of Hope and divided them into two cohorts; cardiac responders as defined by improvement in global longitudinal strain (GLS) over time and cardiac non‐responders as defined by no change or worsening in GLS. We then compared baseline demographics and echocardiographic parameters between the two groups.

**Results:**

There were 16 cardiac responders and 23 cardiac non‐responders identified. Correlation between changes in GLS was compared with change in other echocardiographic parameters. The change in GLS correlated with changes in left ventricular ejection fraction (LVEF) (*r* = −0.44, *p* < 0.01), stroke volume index (*r* = −0.05, *p* < 0.01), posterior wall thickness (*r* = 0.41, *p* = 0.01), right ventricular (RV) free wall strain (*r* = 0.66, *p* < 0.01), right ventricular four‐chamber longitudinal strain (*r* = 0.67, *p* < 0.01), left atrial conduit strain (*r* = 0.46, *p* < 0.01), left atrial reservoir strain (*r* = −0.63, *p* < 0.01), and left atrial contractile strain (*r* = 0.48, *p* < 0.01). At less than 1 year, there was a positive correlation between change in GLS and change in RV free wall strain (*r* = 0.73, *p* < 0.01) and change in right ventricular four‐chamber longitudinal strain (*r* = 0.75, *p* < 0.01).

**Conclusion:**

Improvement in GLS correlated with improvement in RV and left atrial strain measurements in AL‐CA patients receiving treatment. Changes in RV strain were seen early, within 1 year of treatment. Our study suggests that strain imaging in conjunction with cardiac biomarkers can provide a comprehensive assessment of cardiac response.

## Graphical abstract

In patients with AL‐CA undergoing treatment, the incorporation of left atrial and RV strain parameters correlate with left ventricular GLS and predict cardiac response.

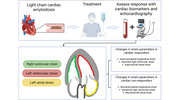

**Abbreviations**
AL: light chain
AL‐CA: light chain cardiac amyloidosis
ATTR‐CM: transthyretin cardiomyopathy
GLS: global longitudinal strain
LA: left atrial
LVEF: left ventricular ejection fraction
NT‐proBNP: N‐terminal Pro‐B‐type Natriuretic Peptide
OS: overall survival
RapLSI: relative apical longitudinal strain index
RV: right ventricular
RV4CLS: four chamber right ventricular longitudinal strain
RVFWSL: right ventricular free wall longitudinal strain
SD: standard deviation
SVI: stroke volume index
TAPSE: tricuspid annular plane systolic excursion
VGPR: very good partial response

## 1 Introduction

Light chain (AL) amyloidosis is a systemic process in which AL complexes infiltrate and disrupt the normal architecture of organs, including the heart [1]. Cardiac involvement is common in AL amyloidosis, and it is not only a leading cause of morbidity and mortality, but also a major determinant of overall survival. AL cardiac amyloidosis (AL‐CA) has several characteristic findings on echocardiography, the main imaging modality used in the evaluation and serial assessment of the disease [2]. Cardiac response to AL‐CA treatment is currently determined by biomarker trends, namely N‐terminal pro‐B‐type natriuretic peptide (NT‐proBNP) [3, 4, 5]. However, NT‐proBNP levels can be drastically altered by filling conditions, such as with acute volume overload, and can also be unreliable in specific populations, such as patients with renal impairment and obesity. These issues may limit the practical use of NT‐proBNP as a serial marker of treatment response. Therefore, the incorporation of imaging markers, which may be less influenced by these factors, should be considered for use in conjunction with NT‐proBNP for overall disease assessment.

Several advanced echocardiographic markers have demonstrated potential benefit in predicting and tracking cardiac response to treatment while correlating with prognosis in AL‐CA^2^. Global longitudinal strain (GLS), which is derived from two‐dimensional speckle tracking, is a measure of longitudinal shortening and has shown promise in the assessment of AL‐CA [6, 7]. GLS is useful for identifying subtle myocardial dysfunction even when left ventricular ejection fraction (LVEF) appears normal, a common finding in AL‐CA. Improvement in GLS has been correlated with cardiac response to treatment and provides prognostic value beyond cardiac biomarkers such as NT‐proBNP [8, 9, 10]. Additionally, speckle tracking offers better reliability and reproducibility compared to other echocardiographic markers such as left ventricular wall thickness and LVEF, rendering it a more precise imaging method for longitudinal evaluation [11]. Other forms of speckle tracking echocardiography may also provide additional information in cases of AL‐CA, including right ventricular (RV) strain and left atrial (LA) strain. While RV involvement may develop later, it has been associated with poor prognosis in AL‐CA [12]. Right ventricular free wall longitudinal strain (RVFWSL) and four chamber right ventricular longitudinal strain (RV4CLS) have both been shown to be useful in assessing RV function and have prognostic value in patients with heart failure [13, 14]. LA function can also be impacted indirectly through diastolic dysfunction as well as directly through amyloid deposition. LA strain has been shown to predict survival in AL‐CA and can be used in the quantitative assessment of LA function in patients with heart failure and amyloidosis [15, 16, 17].

Given emerging data demonstrating that GLS serves as a marker of therapeutic response in AL‐CA, correlates to changes in cardiac biomarkers, and provides incremental prognostic value beyond cardiac biomarkers, we aimed to identify additional echocardiographic strain parameters that correlate with GLS and may help assess cardiac response to treatment.

## 2 Material and methods

This was a retrospective, single‐center study performed at City of Hope. Thirty‐nine patients with AL amyloidosis and known cardiac involvement seen between January 2019 and June 2025 were included. Informed consent was waived as this was a retrospective study. The study conforms to the principles outlined in the Declaration of Helsinki and was performed with the approval of the local institutional review board. Baseline demographics were obtained via retrospective chart review.

Transthoracic echocardiographic images were recorded using GE Vivid E95 (GE Vingmed Ultrasound AS, Horten, Norway) and Philips Epiq CVX (Philips Ultrasound Inc, Bothell, Washington) machines. Echocardiographic data was then digitized and measurements were performed using Tomtec Arena (Philips Medical Systems, Munich, Germany). Two‐dimensional echocardiographic measurements obtained included LVEF, GLS, stroke volume index, average mitral annular s’, interventricular septal thickness, posterior wall thickness, medial and lateral e’, mitral inflow, tricuspid annular plane systolic excursion (TAPSE), RVFWSL, RV4CLS, left atrial volume index, left atrial reservoir strain, left atrial conduit strain, left atrial contractile strain, and estimated right atrial pressure. All studies were performed by multiple operators following a standardized, predefined imaging protocol. Speckle‐tracking myocardial strain imaging was performed using vendor‐based (TomTec Imaging Systems) automated strain calculation software with physician oversight to ensure high fidelity endocardial tracking but with highly limited manual adjustment to minimize inter‐reader variability and optimize reproducibility. Images with suboptimal quality were excluded from this study. Echocardiographic measurements were performed and reviewed by two independent cardiologists. LVEF and stroke volume index were obtained using the modified Simpson method. Relative apical longitudinal strain index (RapLSI) was calculated as (average apical longitudinal strain)/(average basal longitudinal strain + average mid longitudinal strain).

Categorical variables were described using frequencies and percentages, while continuous variables were summarized using means and standard deviations (SD). Differences in baseline demographic and echocardiographic characteristics between cardiac responders and non‐responders were assessed using either chi‐square or Fisher's exact tests for categorical variables and *t*‐tests for continuous variables. Pearson correlation analysis was performed with the change in GLS. Changes in echocardiographic parameters from baseline to follow‐up between the two groups were evaluated using *t*‐tests and multiple linear regression analyses. In the multivariable models, baseline values of the corresponding echocardiographic parameters were included as covariates. Overall survival (OS) by responder group was examined using Kaplan–Meier curves and the log‐rank test in univariate analysis. OS was defined as the time from date of treatment initiation (baseline) to death from any cause and was censored at the most recent visit on or before September 30, 2025, for patients who were still alive. Univariate and multivariable Cox proportional hazards models were used to assess differences in mortality risk by responder group. All statistical tests were two‐sided, and a *p*‐value < 0.05 was considered statistically significant. Data analysis was performed using SAS software, version 9.4 (SAS Institute, Cary, NC).

## 3 Results

A total of 39 patients were divided into two groups; cardiac responders as defined by improvement in GLS versus cardiac non‐responders, or those with no change or worsening in GLS. There were 16 cardiac responders and 23 cardiac non‐responders with a median follow‐up of 3.9 years. All cardiac responders achieved at least very good partial hematologic response (VGPR), defined by a reduction in the difference between involved and uninvolved free ALs to less than 40 mg/L [18]. Of the cardiac non‐responders, 21 patients achieved at least VGPR; the 2 remaining patients had no hematologic response to treatment. The majority of patients were classified as cardiac Mayo stage 1–3 (*n* = 24), the remaining 15 patients were Mayo stage 4 per the revised Mayo 2012 staging system [3]. There was no statistically significant difference in baseline characteristics between groups, as shown in Table 1. Baseline echocardiographic data was compared between both groups, as shown in Table 2. Statistically significant differences in baseline echocardiographic parameters between cardiac responders versus cardiac non‐responders were observed for mean GLS (−10.0 vs. −12.9, *p* = 0.03), mean stroke volume index (23.8 mL/m^2^ vs. 30.0 mL/m^2^, *p* = 0.04), mean E/A ratio (2.18 vs. 1.32, *p* = 0.03), mean left atrial reservoir strain (10.2% vs. 16.5%, *p* = 0.02), mean left atrial conduit strain (−6.1% vs. −8.9%, *p* = 0.04), and mean left atrial contractile strain (−4.3% vs. −8.0%, *p* = 0.03). Otherwise, there was no statistically significant difference in baseline echocardiographic parameters between cardiac responders and non‐responders.

**TABLE 1:** Baseline characteristics.

	Total (*N* = 39)	Cardiac responders (*N* = 16)	Cardiac non‐responders (*N* = 23)	*p* ^*^
Age (yrs), Mean (SD)	62.79 (11.725)	64.19 (13.432)	61.83 (10.586)	0.543
Gender				0.174^†^
Female	12 (30.8%)	7 (43.8%)	5 (21.7%)	
Male	27 (69.2%)	9 (56.3%)	18 (78.3%)	
Race				0.354^†^
Caucasian	23 (59%)	7 (43.8%)	16 (69.6%)	
African American	2 (5.1%)	1 (6.3%)	1 (4.3%)	
Hispanic	8 (20.5%)	4 (25%)	4 (17.4%)	
Asian	6 (15.4%)	4 (25%)	2 (8.7%)	
AL subtype				0.711^†^
Kappa	9 (23.1%)	3 (18.8%)	6 (26.1%)	
Lambda	30 (76.9%)	13 (81.3%)	17 (73.9%)	
Mayo Amyloid Staging				1.000^†^
1	5 (12.8%)	2 (12.5%)	3 (13%)	
2	11 (28.2%)	5 (31.3%)	6 (26.1%)	
3	8 (20.5%)	3 (18.8%)	5 (21.7%)	
4	15 (38.5%)	6 (37.5%)	9 (39.1%)	
Medications				
Beta‐blocker	10 (25.6%)	6 (37.5%)	4 (17.4%)	0.264^†^
Loop diuretic	22 (56.4%)	9 (56.3%)	13 (56.5%)	0.987^†^
Mineralocorticoid receptor antagonist	7 (17.9%)	2 (12.5%)	5 (21.7%)	0.678^†^
ACE inhibitors/ Angiotensin receptor blockers	5 (12.8%)	2 (12.5%)	3 (13%)	1.000^†^
SGLT2 inhibitors	4 (10.3%)	3 (18.8%)	1 (4.3%)	0.286^†^
Aspirin	7 (17.9%)	2 (12.5%)	5 (21.7%)	0.678^†^
Statin	13 (33.3%)	8 (50%)	5 (21.7%)	0.066^†^
Anticoagulation	8 (20.5%)	5 (31.3%)	3 (13%)	0.235^†^
Free light chain ratio, Mean (SD)	21.69 (86.945)	2.01 (5.644)	35.38 (112.062)	0.244
Free light chain (mg/dL), Mean (SD)	183.67 (415.882)	311.65 (626.088)	94.64 (106.224)	0.110
Free light chain difference (mg/dL), Mean (SD)	174.52 (412.150)	296.94 (623.325)	89.36 (100.081)	0.123
Creatinine (mg/dL), Mean (SD)	1.59 (1.292)	2.03 (1.800)	1.29 (0.665)	0.080
NT‐proBNP (pg/mL), Mean (SD)	8226.07 (17257.258)	13692.23 (25226.739)	4046.06 (4433.236)	0.132
B‐type natriuretic peptide (pg/mL), Mean (SD)	582.96 (484.472)	595.58 (542.053)	570.35 (456.916)	0.921
High‐sensitivity troponin I (pg/mL), Mean (SD)	44.30 (49.804)	37.14 (21.264)	60.98 (95.962)	0.520
Troponin I (ng/mL), Mean (SD)	0.14 (0.162)	0.22 (0.254)	0.11 (0.101)	0.130

Abbreviations: NT‐proBNP, N‐terminal pro‐B‐type natriuretic peptide; SD, standard deviation; SGLT2 inhibitors, sodium‐glucose cotransporter 2 inhibitors.

Continuous variables are displayed as mean (SD). Categorical variables were reported frequency count and percentage of patients.

*p* ascertained from *t*‐test, except where otherwise noted.^†^ *p* ascertained from chi‐square or Fisher's exact test whichever is appropriate.

**TABLE 2:** Mean (SD) of baseline echocardiographic parameters.

		Mean (SD) by cardiac responder		
	Mean (SD) (*N* = 39)	Responders (*N* = 16)	Non‐responders (*N* = 23)	*p*
GLS (%)	−11.72 (4.12)	−10.00 (3.17)	−12.92 (4.33)	0.027
LVEF (%)	56.34 (7.88)	56.14 (8.99)	56.48 (7.21)	0.896
Stroke volume index (mL/m^2^)	27.57 (9.39)	23.78 (6.80)	30.04 (10.13)	0.043
Average mitral annular s’ (m/s)	0.06 (0.02)	0.06 (0.02)	0.06 (0.03)	0.897
Interventricular septal thickness (mm)	14.37 (1.90)	14.49 (1.81)	14.28 (1.99)	0.741
Posterior wall thickness (mm)	14.31 (2.12)	14.23 (1.91)	14.36 (2.30)	0.850
Medial e’ (m/s)	0.05 (0.02)	0.05 (0.01)	0.05 (0.02)	0.239
Lateral e’ (m/s)	0.07 (0.03)	0.06 (0.02)	0.07 (0.03)	0.239
E/e’ ratio	18.48 (9.56)	20.60 (9.87)	16.79 (9.21)	0.240
E/A ratio	1.66 (1.12)	2.18 (1.18)	1.32 (0.96)	0.028
TAPSE (cm)	1.81 (0.44)	1.69 (0.30)	1.89 (0.50)	0.196
RVFWSL (%)	−18.00 (6.63)	−16.90 (6.43)	−18.76 (6.80)	0.396
RV4CSL (%)	−13.47 (5.22)	−12.64 (5.14)	−14.05 (5.31)	0.414
Left atrial volume index (ml/m^2^)	37.37 (9.75)	38.47 (7.72)	36.65 (10.98)	0.579
Left atrial reservoir strain (%)	13.91 (6.47)	10.20 (6.47)	16.49 (8.81)	0.020
Left atrial conduit strain (%)	−7.76 (4.27)	−6.09 (2.93)	−8.91 (4.72)	0.041
Left atrial contractile strain (%)	−6.48 (5.39)	−4.33 (4.76)	−7.99 (5.38)	0.035
Apical longitudinal strain (%)	−15.24 (4.24)	−13.86 (4.24)	−16.20 (4.06)	0.090
Medial longitudinal strain (%)	−10.09 (4.60)	−8.42 (3.83)	−11.25 (4.80)	0.057
Basal longitudinal strain (%)	−8.76 (5.28)	−6.75 (4.48)	−10.16 (5.44)	0.046
RapLSI	0.96 (0.44)	1.04 (0.39)	0.91 (0.47)	0.388
Time between echocardiograms (days)	765.26 (748.19)	447.13 (334.69)	986.57 (875.05)	0.025

Abbreviations: SD, standard deviation; GLS, global longitudinal strain; LVEF, left ventricular ejection fraction; TAPSE, tricuspid annular plane systolic excursion; RVFWSL, right ventricular free wall longitudinal strain; RV4CSL, four chamber right ventricular longitudinal strain; RapLSI, relative apical longitudinal strain index.

All values are reported as Mean (SD). *p* ascertained from *t*‐test.

**FIGURE 1:**
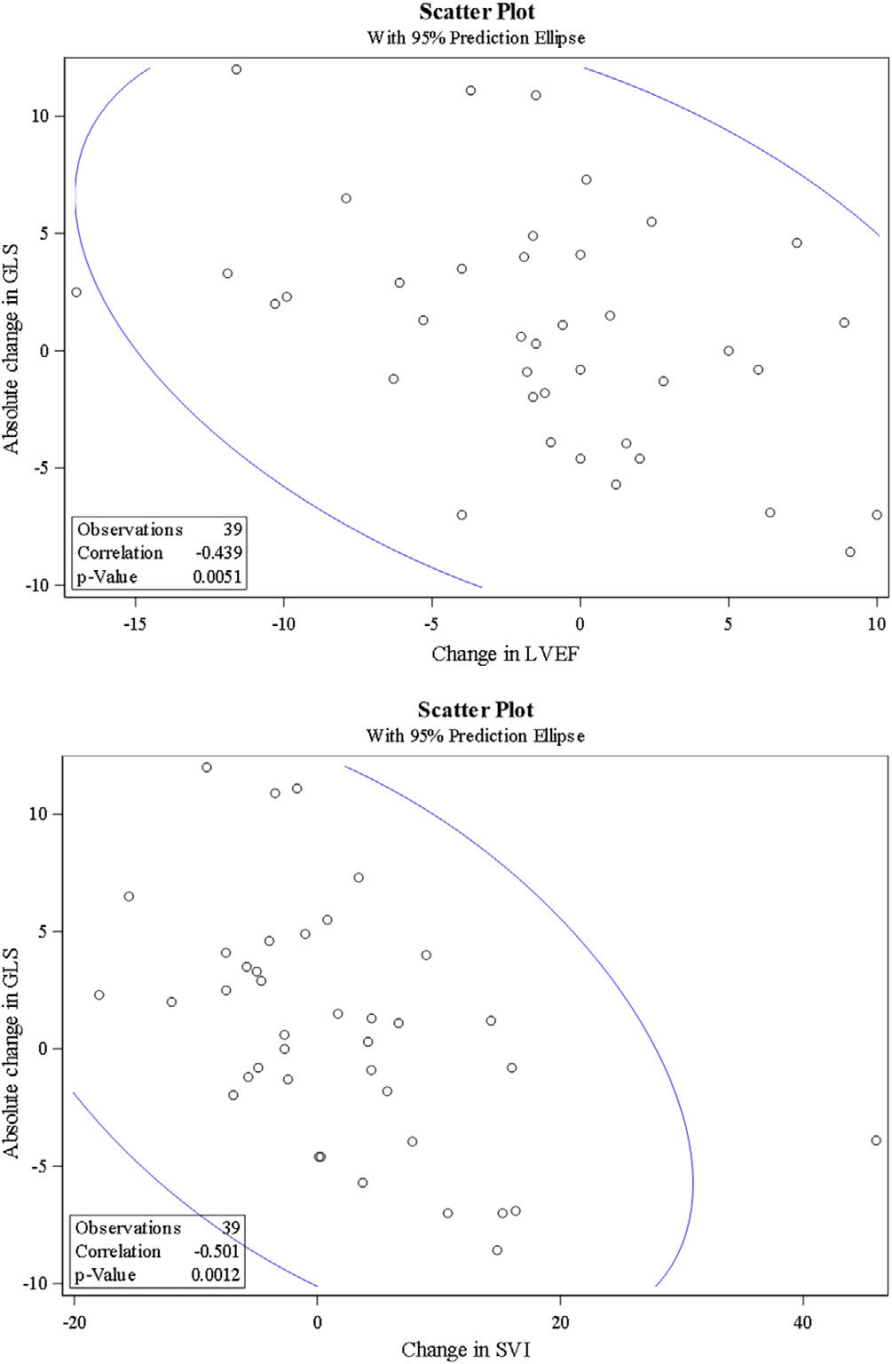
Correlation between change in GLS and left ventricular systolic function. (A) Correlation between change in GLS and change in LVEF. (B) Correlation between change in GLS and change in stroke volume index (SVI). Correlation reported using Pearson correlation coefficient.

**FIGURE 2:**
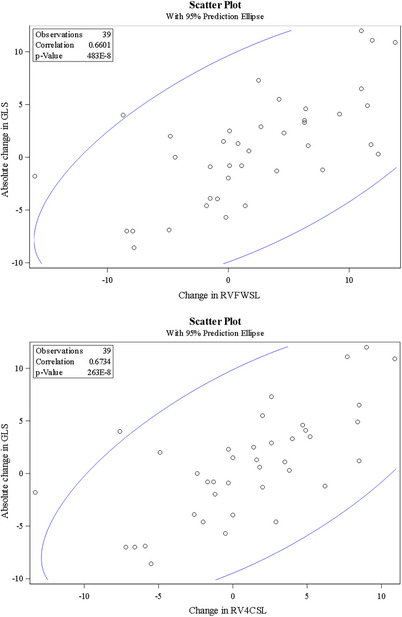
Correlation between changes in GLS and RV strain. (A) Correlation between change in GLS and change in RVFWSL. (B) Correlation between change in GLS and change in right ventricular 4‐chamber longitudinal strain (RV4CSL). Correlation reported using Pearson correlation coefficient.

**FIGURE 3:**
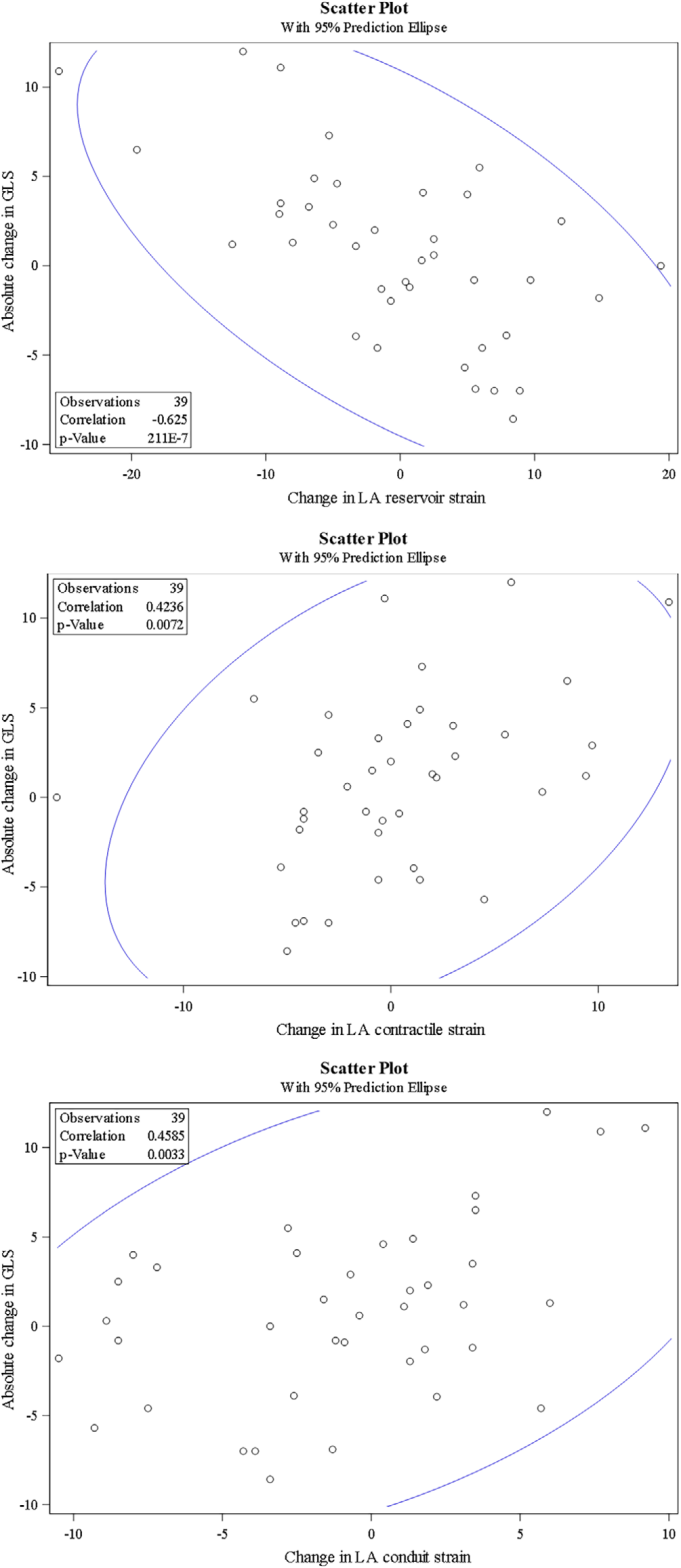
Correlation between change in GLS and left atrial strain. (A) Correlation between change in GLS and change in left atrial reservoir strain. (B) Correlation between change in GLS and change in left atrial contractile strain. (C) Correlation between change in GLS and change in left atrial conduit strain. Correlation reported using Pearson correlation coefficient.

Correlation between change in GLS and change in other echocardiographic markers was then assessed, as shown in Table 3 and Figures 1, 2, 3. The change in GLS was negatively correlated with changes in LVEF (*r* = −0.44, *p* < 0.01), stroke volume index (*r* = −0.05, *p* < 0.01), and left atrial reservoir strain (*r* = −0.63, *p* < 0.01). In contrast, change in GLS was positively correlated with changes in posterior wall thickness (*r* = 0.41, *p* = 0.01), RV free wall strain (RVFWSL; *r* = 0.66, *p* < 0.01), RV four‐chamber longitudinal strain (RV4CLS; *r* = 0.67, *p* < 0.01), left atrial conduit strain (*r* = 0.46, *p* < 0.01), and left atrial contractile strain (*r* = 0.48, *p* < 0.01).

**TABLE 3:** Correlation with absolute change in GLS.

	Overall (*N* = 39)	Patients with follow‐up time < 1 year (*N* = 19)	Patients with follow‐up time ≥ 1 year (*N* = 20)
Change in LVEF (%)	−0.44 (0.005)	−0.49 (0.034)	−0.38 (0.095)
Change in stroke volume index (mL/m^2^)	−0.50 (0.001)	−0.56 (0.013)	−0.49 (0.03)
Change in interventricular septal thickness (mm)	0.30 (0.06)	0.15 (0.54)	0.49 (0.03)
Change in posterior wall thickness (mm)	0.41 (0.01)	0.46 (0.049)	0.37 (0.112)
Change in RVFWSL (%)	0.66 (<.0001)	0.73 (0.0004)	0.65 (0.002)
Change in RV4CSL (%)	0.67 (<.0001)	0.75 (0.0002)	0.66 (0.001)
Change in left atrial reservoir strain (%)	−0.63 (<.0001)	−0.44 (0.060)	−0.76 (<.0001)
Change in left atrial conduit strain (%)	0.46 (0.003)	0.29 (0.227)	0.61 (0.005)
Change in left atrial contractile strain (%)	0.48 (0.002)	0.27 (0.263)	0.64 (0.002)

Abbreviations: LVEF, left ventricular ejection fraction; RVFWSL, right ventricular free wall longitudinal strain; RV4CSL, four chamber right ventricular longitudinal strain.

Pearson correlation coefficients (*p*) are reported*. P* ascertained from *t*‐test.

In the 19 patients with less than 1 year of follow‐up, there remained a negative correlation between change in GLS and change in LVEF (*r* = −0.49, *p* = 0.03) and change in GLS and change in stroke volume index (*r* = −0.56, *p* = 0.01). There was still a positive correlation between change in GLS and change in RVFWSL (*r* = 0.73, *p* < 0.01) and change in GLS and change in RV4CSL (*r* = 0.75, *p* < 0.01).

The mean changes in echocardiographic parameters in cardiac responders and non‐responders are shown in Table 4. Cardiac responders had a mean change in GLS of −3.81% while cardiac non‐responders had a mean change in GLS of 4.06% (*p* < 0.01). There was also a concurrent significant difference in the change in LVEF, LV wall thickness, RVFWSL, RV4CSL, and left atrial reservoir strain between the two groups on multivariate analysis after adjusting for baseline values of echocardiographic parameters.

**TABLE 4:** Mean change (SD) of echocardiographic parameters.

	Total (*N* = 39)	Cardiac responders (*N* = 16)	Cardiac non‐responders (*N* = 23)	Unadjusted *p*	Adjusted *p*
Change in GLS (%)	0.83 (5.01)	−3.81 (2.64)	4.06 (3.47)	<0.0001	<0.0001
Change in LVEF (%)	−1.25 (6.04)	1.45 (4.52)	−3.13 (6.33)	0.018	0.011
Change in stroke volume index, (mL/m^2^)	1.68 (11.17)	7.59 (12.97)	−2.43 (7.61)	0.004	0.054
Change in mitral annular s' (m/s)	−0.004 (0.03)	−0.003 (0.02)	−0.004 (0.03)	0.851	0.078
Change in IVS thickness (mm)	−0.39 (2.64)	−1.40 (2.31)	0.31 (2.68)	0.045	0.047
Change in posterior wall thickness (mm)	−0.38 (2.32)	−1.28 (2.48)	0.24 (2.03)	0.042	0.020
Change in medial e' (m/s)	0.00 (0.03)	0.00 (0.02)	−0.01 (0.03)	0.273	0.027
Change in lateral e' (m/s)	0.00 (0.04)	0.01 (0.02)	−0.01 (0.05)	0.244	0.182
Change in E/e'	0.73 (12.33)	−3.08 (9.94)	3.37 (13.32)	0.109	0.723
Change in E/A	−0.23 (1.48)	−0.29 (1.18)	−0.19 (1.68)	0.843	0.116
Change in TAPSE (cm)	−0.06 (0.79)	0.18 (0.77)	−0.23 (0.77)	0.105	0.246
Change in RVFWSL (%)	2.05 (6.92)	−2.28 (5.65)	5.07 (6.15)	0.0005	0.0006
Change in RV4CSL (%)	1.00 (5.31)	−2.31 (4.62)	3.30 (4.54)	0.0006	0.0005
Change in left atrial volume index (ml/m^2^)	3.92 (13.29)	5.17 (14.00)	3.05 (13.02)	0.631	0.998
Change in left atrial reservoir strain (%)	−0.36 (9.05)	4.54 (5.05)	−3.77 (9.71)	0.003	0.028
Change in left atrial conduit strain (%)	−0.89 (5.05)	−2.44 (4.76)	0.19 (5.07)	0.111	0.458
Change in left atrial contractile strain (%)	0.51 (5.26)	−1.88 (2.88)	2.18 (5.92)	0.016	0.125
Change in RapLSI	0.00 (0.38)	−0.07 (0.40)	0.05 (0.36)	0.328	0.5552

Abbreviations: SD, standard deviation; GLS, global longitudinal strain; LVEF, left ventricular ejection fraction; IVS, interventricular septal; TAPSE, tricuspid annular systolic excursion; RVFWSL, right ventricular free wall longitudinal strain; RV4CSL, four chamber right ventricular longitudinal strain; RapLSI, relative apical longitudinal strain index.

Change of echocardiographic parameters are reported as Mean (SD). Unadjusted *p* ascertained from *t*‐test. Adjusted *p* ascertained from multivariable linear regression model, in which baseline values of the corresponding echocardiographic parameters were included as covariates.

**FIGURE 4:**
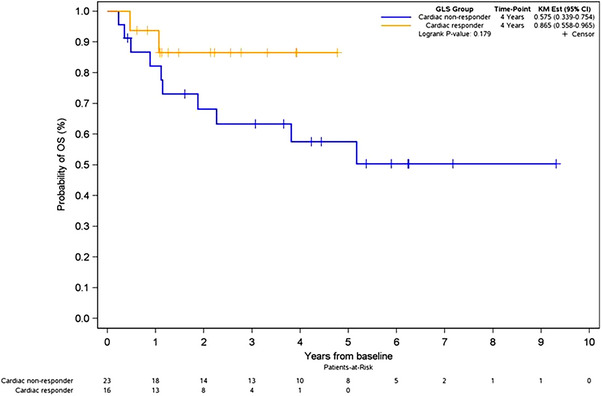
Kaplan–Meier curve for overall survival, as stratified by cardiac responders and cardiac non‐responders.

Of the 16 cardiac responders, 2 passed away during follow‐up (12%). Of the 23 cardiac non‐responders, 10 passed away during follow‐up (43%). Kaplan–Meier analysis demonstrated a trend towards lower all‐cause mortality in cardiac responders, although this was not statistically significant (*p* = 0.179; Figure 4).

## 4 Discussion

Cardiac response to AL‐CA treatment is currently monitored through changes in NT‐proBNP levels, however exclusive reliance on this biomarker is problematic as conditions like renal dysfunction, obesity, and acute volume overload may confound its interpretation [19]. Renal involvement is exceedingly common in AL amyloidosis and can lead to persistently elevated biomarker levels, which in turn may mask cardiac response to treatment. Incorporation of echocardiographic imaging parameters may provide a more accurate longitudinal assessment of cardiac response to treatment. Strain imaging by speckle tracking is becoming increasingly popular, can be performed rapidly, and serves as a more sensitive assessment of myocardial function. Strain imaging has been shown to be useful in the diagnosis and monitoring of AL‐CA. Speckle tracking echocardiography has several advantages in assessing cardiac function over traditional measurements such as LVEF and Doppler measurements, including greater reproducibility, less angle dependence, and less sensitivity to loading conditions and volume [11]. There is growing evidence that treatment response in AL‐CA can be seen through changes in GLS with good correlation to changes in biomarker levels [2, 7, 10]. An absolute improvement in GLS by −2% in patients with AL‐CA was found to be associated with increased overall survival independent of biomarker response [10].

Given the known correlation between changes in cardiac biomarkers and GLS, we used GLS to define cardiac responders and non‐responders [7]. At baseline, there was no difference in LVEF and stroke volume between cardiac responders and non‐responders. Our study showed that improvement in GLS was correlated with a concurrent increase in LVEF and stroke volume index, further supporting an improvement in overall cardiac function. Furthermore, there was a non‐significant trend towards a reduction in all‐cause mortality for cardiac responders, which is in line with the concept that cardiac responders have improved overall survival [19].

While the diagnosis and monitoring of AL‐CA is typically focused on LV parameters, recent literature has suggested that speckle tracking strain imaging of all cardiac chambers may have important prognostic value [15]. RV strain has been associated with mortality risk, and a decline in RVFWSL over time has been correlated with a higher risk of all‐cause mortality and heart failure hospitalization [20, 21]. Assessment of RV function by conventional parameters such as TAPSE and tricuspid S’ wave peak systolic velocity are angle and load‐dependent, making them less reliable as longitudinal markers [22]. Conversely RV strain parameters have shown better reproducibility and sensitivity for detecting subclinical RV dysfunction and may be a better imaging marker for serial assessment [22]. Our study showed that AL‐CA patients with cardiac response also demonstrated improvement in RVFWSL and RV4CL. In cardiac responders, RVFWSL and RV4CSL each had improvement by a mean of 2.3% while non‐responders had a decrease in RVFWSL by 5.1% and RV4CSL by 3.3%. In fact, these improvements were seen within one year of therapy, indicating that RV strain may be useful as an early marker of cardiac response. While this has not been demonstrated in AL‐CA, prior studies have shown improvement in RVFWSL in patients with transthyretin cardiomyopathy (ATTR‐CM) treated with tafamidis, which supports the use of speckle tracking strain imaging of the right ventricle in cardiac amyloidosis [23, 24]. Future studies need to be done to identify a cut‐off value for predicting cardiac response.

Atrial dysfunction in cardiac amyloidosis is being increasingly recognized as an important parameter in the diagnosis and monitoring of disease. Histopathological data suggests that atrial involvement may even precede LV dysfunction, highlighting the importance of monitoring atrial function [25]. LA strain has been shown to predict overall survival in AL‐CA [15]. While studies assessing atrial response to treatment are limited at this time, LA strain has been shown to improve in ATTR‐CM treated with tafamadis [16], which supports the use of this parameter to monitor response to treatment in ATTR‐CM and potentially AL‐CA as well [15]. Our results demonstrated a significant moderate correlation between improvements in GLS with improvement in LA strain, further supporting the utility of serial LA strain assessment in patients undergoing treatment. We showed a correlation in all three LA strain parameters (reservoir, conduit and contractile), which is important as contractile strain may be inaccurate in patients with atrial fibrillation, which is commonly seen in cardiac amyloidosis [25]. LA conduit and reservoir strain maintain their utility regardless of rhythm, and we showed that they too demonstrate improvement with cardiac response to treatment. In our study population, left atrial reservoir strain and conduit strain improved by 4.5% and 1.9% respectively in cardiac responders while non‐responders showed worsening of these parameters.

In our study, cardiac responders appeared to have more significant baseline cardiac impairment, as demonstrated by lower LV and LA strain and more advanced diastolic dysfunction. Our findings differ from prior studies that have shown that patients with more advanced cardiac amyloidosis have poorer prognosis and less favorable cardiac response. In our study, all patients achieved at least very good partial hematologic response, as opposed to prior studies that have included varying degrees of hematologic response. It is possible that deep hematologic response plays a more significant role than baseline cardiac dysfunction in determining cardiac response to therapy and overall prognosis. A study done by Muchtar et al. showed that a profound hematologic response improves the odds of a meaningful cardiac response, which subsequently increases overall survival, regardless of cardiac staging [26]. However in patients not attaining complete hematologic remission, more advanced cardiac stage was associated with poorer survival. These findings parallel our findings and suggest that hematologic response is crucial in determining cardiac response and survival. Patients with more severe baseline cardiac impairment may also exhibit greater dynamic range for improvement in echocardiographic markers despite only achieving a modest improvement in the absolute value of studied measurements. Another consideration is that differential hematologic treatment intensity often occurs in real‐world practice, where patients with worse cardiac involvement may receive more aggressive or earlier therapy, closer monitoring, or faster escalation in treatment. Ultimately, due to our small sample size and retrospective nature of the study, we cannot establish definitive conclusions about our results and propose that our findings serve to generate hypotheses for future prospective studies.

Our study has several limitations. This was a single‐center retrospective study with a small sample size, due to the overall rarity of AL‐CA. We did not have follow‐up cardiac biomarkers available for all patients, limiting our ability to assess the association of biomarker change with echocardiographic parameters. The majority of our patients achieved at least very good partial hematologic response, therefore we were unable to examine the difference in echocardiographic parameters in those with and without hematologic response. Given the retrospective nature of the study, we are unable to correlate our findings with clinical outcomes.

## 5 Conclusions

In conclusion, improvement in GLS in AL‐CA patients receiving treatment correlated with improvement in RV and LA strain measurements. The changes in RV strain were seen early, within less than 1 year of treatment. Our study suggests that strain imaging by speckle tracking can be used routinely in conjunction with cardiac biomarkers to monitor cardiac response to therapy in patients with AL amyloidosis, and these imaging parameters may be more reliable and less impacted by other patient factors. We also demonstrated a trend towards improved survival in cardiac responders, but further studies are needed to confirm whether improvements in echocardiographic markers correlate with improved clinical outcomes.

## Funding

This research did not receive any specific grant from funding agencies in the public, commercial, or not‐for‐profit sectors.
